# Flagellotropic Bacteriophages: Opportunities and Challenges for Antimicrobial Applications

**DOI:** 10.3390/ijms23137084

**Published:** 2022-06-25

**Authors:** Nathaniel C. Esteves, Birgit E. Scharf

**Affiliations:** Virginia Tech Department of Biological Sciences, Blacksburg, VA 24061, USA; nesteves@vt.edu

**Keywords:** phage, flagellum-dependent, phage therapy, flagella, motility, pathogens, virulence

## Abstract

Bacteriophages (phages) are the most abundant biological entities in the biosphere. As viruses that solely infect bacteria, phages have myriad healthcare and agricultural applications including phage therapy and antibacterial treatments in the foodservice industry. Phage therapy has been explored since the turn of the twentieth century but was no longer prioritized following the invention of antibiotics. As we approach a post-antibiotic society, phage therapy research has experienced a significant resurgence for the use of phages against antibiotic-resistant bacteria, a growing concern in modern medicine. Phages are extraordinarily diverse, as are their host receptor targets. Flagellotropic (flagellum-dependent) phages begin their infection cycle by attaching to the flagellum of their motile host, although the later stages of the infection process of most of these phages remain elusive. Flagella are helical appendages required for swimming and swarming motility and are also of great importance for virulence in many pathogenic bacteria of clinical relevance. Not only is bacterial motility itself frequently important for virulence, as it allows pathogenic bacteria to move toward their host and find nutrients more effectively, but flagella can also serve additional functions including mediating bacterial adhesion to surfaces. Flagella are also a potent antigen recognized by the human immune system. Phages utilizing the flagellum for infections are of particular interest due to the unique evolutionary tradeoff they force upon their hosts: by downregulating or abolishing motility to escape infection by a flagellotropic phage, a pathogenic bacterium would also likely attenuate its virulence. This factor may lead to flagellotropic phages becoming especially potent antibacterial agents. This review outlines past, present, and future research of flagellotropic phages, including their molecular mechanisms of infection and potential future applications.

## 1. Introduction

### 1.1. Phages among Us

With an estimated total population of 10^31^, bacteriophages are by far the most abundant biological entities on earth, more than all others combined [1]. Within their diversity lies one of phages’ greatest strengths as potential antibacterial agents: a bacteriophage-based treatment can be specifically tailored to an individual organism [2,3], avoiding disruption of the natural bacterial flora. While a physician proposing the use of viruses as beneficial therapeutic agents to patients can be seen as suspicious to the layman, broadening knowledge in the field of phages will likely improve public opinion regarding phage therapy.

The majority of discovered bacteriophages and the vast majority of well-studied ones belong to the order *Caudovirales* [4], known also as tailed dsDNA phages encoding the HK97-fold major capsid protein [5]. This order is subdivided into three major families: *Myoviridae* (long contractile tails), *Siphoviridae* (long non-contractile tails), and *Podoviridae* (short non-contractile tails), all of which have distinct structural characteristics [4]. All of the phages discussed in this review belong to one of these three families.

### 1.2. Broader Applications of the Viruses of Bacteria

Phage therapy is far from a new concept [6,7,8]. Frederick Twort and Felix d’Herelle are both credited with discovering phages independently of each other in 1915 and 1917, respectively. While Twort was unsure about the nature of the entity causing bacterial lysis, d’Herelle correctly characterized them as viruses parasitizing bacteria [7]. Immediately after their discovery near the turn of the 20th century, phages were used to treat bacterial infections [9]. d’Herelle himself cured bacterial dysentery multiple times using a phage treatment. Later, d’Herelle, alongside George Eliava, successfully employed phage treatments against *Vibrio cholerae* and *Yersinia pestis* [7]. Phage therapy was utilized for years, but with the discovery and popularization of antibiotics a few decades later, phages were largely forgotten as therapeutics [10]. As rates of antibiotic-resistant bacterial infections continue to rise precipitously, phage therapy research has regained its popularity [11,12,13,14,15]. Antibiotic resistance is a growing problem, with many multi-drug resistant (MDR) bacteria emerging every year, including the appearance of so called “pan-resistant” strains, which are resistant to all or nearly all antibiotics on the market [16,17,18,19]. A major downside of antibiotics when compared to phages is their limited options. While the number of antibiotics on the market is a large but finite number, the number of phages in the world is practically infinite [1,20,21]. The fact that most phages are discovered in locations such as bodies of water, sewage, and infected animals is a testament to the number of new phages that can be found in these niches, which can potentially be isolated and applied in a useful context. The self-replicating nature of phages makes their isolation simple and straightforward [22,23], with propagation being fairly trivial as well [24]. Their remarkable host specificity is an additional benefit, although this may also pose a drawback for broad-spectrum therapies [25,26]. Upon identification of the bacterial pathogen causing an infection, a tailored phage treatment can be designed to target solely this species or serotype. However, host–phage specificity requires that the pathogenic species has been identified before treatment can be started. Antibiotics do not have this issue as broad-spectrum compounds are available [27,28,29]. This comes at a cost: broad-spectrum drugs such as the carbapenem class of antibiotics are known for their side effects, mostly due to the elimination of the natural bacterial flora [30,31,32]. Disruption of the delicate balance of organisms that live within us can lead to superinfections, where a single pathogen can take over following antibiotic treatment [33,34,35]. A tailored phage treatment would not eliminate natural flora, as phage specificity could avoid killing beneficial organisms. Phages can also be genetically engineered to exhibit broad spectrum antimicrobial activity [26,36,37,38,39], as this is necessary for the swift treatment of unknown and potentially lethal infections. Phage therapy is legal and commonly employed in several countries, including Georgia, Poland, and Russia [40], while it can only be used as a last resort in other countries including the United States, Australia, and a number of western European nations [12,40].

Another applied use for phages is in the foodservice industry, where they are frequently used to protect produce from spoilage and to clean surfaces as a preventive measure against particularly virulent pathogenic bacteria. Candidates for this application include pathogens such as *Listeria monocytogenes*, *Shigella dysenteriae*, Shiga-toxigenic *Escherichia coli*, *Salmonella enterica* serotype Typhi, and *Clostridium botulinum* [41,42,43]. Since phage-based products used in the foodservice industry are not used directly in live humans, these products are not subject to the strict FDA requirements that is a current roadblock to phage therapy [44].

### 1.3. An Exploitable Evolutionary Tradeoff

An evolutionary tradeoff for bacteria refers to a stressful condition that cannot be easily avoided without introducing a different stressful condition [45,46,47,48,49]. Perhaps the most straightforward example in phage biology is infection via an antibiotic-resistance complex, such as an efflux pump. For instance, phage OMKO1 infects *Pseudomonas aeruginosa* via a multi-drug efflux system that mediates MDR [46]. The simplest way for the bacterium to develop resistance to OMKO1 is by repression of antibiotic pump gene transcription. This leads to reduced prevalence of the complex and thus results in the phage having more difficulty infecting the cells. If the efflux system mutates to become entirely non-functional, the mutant cell is completely resistant to OMKO1. The tradeoff arises from the fact that by downregulating or mutating this multi-drug efflux pump, the bacterial cells become more susceptible to the substrates of the pump. In the case of OMKO1, this factor has been found to be exploitable by introducing the phage and antibiotic simultaneously [46,50].

The tradeoff for flagellotropic phages is quite a bit simpler. Flagellar motility is a crucial virulence factor for most motile pathogens, and abolishment of motility results in the partial or sometimes complete attenuation of many organisms [51,52,53,54,55]. When a flagellotropic phage is present, there is huge selective pressure for the bacterial cells to repress motility. If motility is abolished, they become completely resistant to the phage, posing a threat. However, this has the unintended and exploitable side effect of reducing virulence. Flagellotropic phages that additionally utilize other virulence factors as secondary receptors may be of particular interest.

## 2. The Flagellotropic Phage Niche

### 2.1. Phages and Their Myriad Host Receptors

The infection processes of different phages are complex and distinct from one another. However, all phages must begin their infection by attaching to a receptor [56]. Bacterial phage receptors are as diverse as the viruses themselves, as are the receptor binding proteins (RBPs) produced by the phages [57,58]. A receptor is a cellular component that a phage utilizes to attach to the cell. The receptor can also serve as a mechanism for ejection of viral DNA into its host, although this is not always the case [59,60,61,62]. It behooves a phage to utilize a receptor that is indispensable, or at least important, for its host [48,63]. Otherwise, all a host cell must do to develop resistance is to no longer express the receptor that is being hijacked by the phage [63,64]. It is for this reason that phages targeting crucial cellular components or virulence factors are of particular interest for antimicrobial applications. Examples of virulence factors hijacked by bacteriophages include antibiotic efflux systems [46,65], capsules [66], flagella [67], lipopolysaccharide (LPS) [66,68], and pili [67].

### 2.2. The Bacterial Flagellum

The bacterial flagellum is a corkscrew-shaped appendage that is responsible for bacterial swimming and swarming motility, during which the flagellum rapidly rotates at speeds of greater than 1000 Hz in genera such as *Vibrio*, with 100–300 Hz being a more typical rotation speed for most bacterial species [69]. In the archetypal flagellar assembly conserved across multiple phyla, the flagellar motor is a nanomachine powered by the proton motive force (PMF) or an Na^+^ ion gradient, which allows the cells to travel many cell-lengths per second [69,70,71]. The *E. coli* motor is bi-directional, capable of counterclockwise (CCW) and clockwise (CW) rotation [72,73,74]. When flagella rotate CCW, they form a tight flagellar bundle that propels the cell in a straight direction known as a run [73,74,75]. When any of the bundled flagella rotate CW, the bundle falls apart, and the cell undergoes a tumble, during which it reorients itself randomly, altering its swimming direction. In contrast, some species of bacteria such as the alfalfa symbiont *Sinorhizobium* (*Ensifer*) *meliloti* have a unidirectional speed-variable motor that functions similarly; a sharp decrease in rotational speed, rather than switching the direction of rotation, results in a tumble [76]. Bacteria have mechanisms for sensing their environment, as it benefits them greatly to move toward nutrients and away from potential repellents. This is accomplished through a complex system known as chemotaxis, which allows bacterial cells to sense attractants or repellants in their environment via chemoreceptors and respond appropriately by biasing the rotational direction or speed of the flagellum [77,78,79,80,81]. In addition to motility, effective chemotaxis are important virulence factors for many species of bacteria [52,53].

The flagellum is composed of three main structures: the basal body, the flexible hook, and the corkscrew-shaped filament [54,70,82,83]. The basal body serves as a structural anchor, polymerization platform, and secretion channel, and is required for torque generation and rotation. The filament is the “propeller” which pushes the bacterium through the medium, and the hook is the joint that connects the filament to the basal body [54,84]. Different species of bacteria synthesize diverse numbers of flagella and organize them differently on the cell. Some produce numerous flagella, while others produce a single flagellum. Some bacteria produce either one flagellum at one pole or one at each pole (polar) [85], and distribute them randomly on the cell surface (peritrichous) [85] or in a single or multiple clumps (lophotrichous) [86]. Flagellotropic bacteriophages are diverse but share the common trait of attachment to the filament of the flagellum being their initial infection step [87,88]. The flagellar filament is composed of monomers of flagellin [54,89]. These monomers polymerize into a helical pattern, forming the filament [90]. A single filament may contain up to 30,000 individual flagellin monomers [90] and can be 10–15 μm in length [54,90], many times the length of the cell itself. The flagellin structure is highly variable yet shares important similarities across species [90]. Flagellin is typically composed of seven domains: two D0, D1, and D2 domains on the C- and N-terminal ends, and a hypervariable D3 domain in the center [89,90]. When flagellin monomers are assembled into the flagellum, the D2 domains and the D3 domain face outward and form the major antigenic region, while the other domains are more conserved and interact with one another to form the filament [89]. Even within a given species, domains may have very low sequence conservation between strains and serotypes, can vary in length by up to 1000 amino acid residues, or even be absent altogether [90]. Flagellin is also frequently modified post-translationally in various bacterial species. Types of modifications include glycosylation [91,92,93] and methylation [94]. There is also the important distinction between sheathed and unsheathed flagella. The sheath refers to a section of membrane that is wrapped around the entire flagellar filament [95,96]. In organisms with sheathed flagella, the flagellar filament does not pierce the outer membrane of the cell; instead, the membrane is secured at the base of the filament by a ring and envelops the entire flagellum [95,96].

### 2.3. Distinct Advantages of the Flagellotropic Lifestyle

Apart from the aforementioned evolutionary tradeoff, there are other advantages afforded to phages that utilize flagella as their receptors. Flagellar motility is a very costly process for the bacterial cell. The motor consistently utilizes the PMF generated by proton export from cellular metabolic processes [97,98,99]. This takes away from PMF energy that could otherwise be used for other crucial cellular functions. For this reason, flagellum production and function are very tightly regulated [74,100,101,102]. Most bacteria alter the expression of flagellar components in response to their environment. Factors such as reduced nutrient availability [103] and sub-optimal temperatures [104] frequently cause repression of motility, as the cell must save its energy for more essential processes for survival. Cells will generally not be motile if it would cause a significant fitness deficit to do so. For this reason, the flagellotropic phage lifestyle specifically selects for hosts that are ideal for viral replication. A cell with a fitness deficit that would result in reduced viral replication would also likely be non-motile. This reduces the likelihood that a flagellotropic phage will inadvertently infect a sub-optimal host.

Since phages have no motility, interaction with their hosts must occur randomly. The flagellum is a large appendage, frequently many times the length of the cell itself [90]. Therefore, the target for flagellotropic phages is much larger than for most other phages, such as those that use LPS or an outer membrane channel as receptors.

## 3. Host Bacteria and Their Respective Flagellotropic Phages of Study

Numerous flagellotropic phages have been discovered, but the level of cumulative knowledge of these phages varies. Below, we discuss individual flagellum-dependent phages. These phages are sorted by their relative depth of study and are summarized in Table 1. In this review, we attempted to give a complete overview of flagellum-dependent phages, but apologize if any data has been overlooked.

### 3.1. Salmonella Phage χ

Salmonella is a ubiquitous genus of Gram-negative enteric bacteria, well known for causing gastroenteritis associated with foods such as raw chicken [105,106]. Salmonella is the most common infectious agent triggering bacterial gastroenteritis [106]. In addition, certain Salmonellae can be highly virulent and invasive [107,108], potentially reaching the bloodstream from the intestine and resulting in deadly bacteremia. S. enterica is a very broad species, containing over 2500 individual serotypes [109], each with distinct phenotypes. These include serotypes such as Typhimurium, Enteritidis, and Newport, each of which cause gastroenteritis in humans but can be commensal in livestock. This is in contrast to serotype Typhi, a highly adapted human-specific pathogen causing Typhoid fever [110]. Salmonella is also the most comprehensively sequenced bacterial genus [111]. Many flagellotropic phages infecting Salmonella have been found, including the archetypal phage χ [112].

Bacteriophage χ (sometimes referred to as ΦΧ prior to 1967 [112]) is probably the most well characterized flagellotropic phage. Bacteriophage χ was the first phage determined to be flagellum dependent, infecting multiple genera of Enterobacterales. Sertic and Boulgakov discovered χ in 1936 as a phage specific to flagellated bacteria [113]. Phage χ was then characterized much more in-depth in the 1960s by Elinor Meynell and Julius Adler [87,112,114]. Its most frequently studied host is S. enterica, of the family Enterobacteriaceae. S. enterica is an incredibly diverse species, and only some of its many serotypes are susceptible to phage χ. The closely related Enterobacteriaceae organism E. coli is also infected by χ [87], and this is also strain-dependent. Lastly, the more distantly related organism Serratia marcescens of the Yersiniaceae family is a host for χ [115]. Due to the diversity of its host range, it is certainly possible that χ may be capable of infecting other species of bacteria. This virulent Siphoviridae phage uses its approximately 220-nm-long tail fiber to attach to the host’s flagellum by wrapping the fiber around the filament and using rotation to translocate to the cell surface [87,116]. As a siphophage, χ has a long (~230 nm), non-contractile tail and an icosahedral capsid with a diameter of approximately 66 nm [117]. It has a 59,407 bp genome with 75 open reading frames [117], most of which have no annotated function. The tail fiber protein likely serves as an RBP for attachment to the main receptor, the flagellum. The flagellum is one of the main differences between the unique serovars of Salmonella [109], so subtle variations in flagellar structure likely contribute to phage χ’s selective host range. It is thought that the multi-substrate efflux system AcrAB/TolC serves as a secondary receptor for χ [65].

### 3.2. Bacillus Phage PBS1

*Bacillus* is a very broad genus of Gram-positive, rod-shaped, spore-forming, aerobic or facultatively anaerobic bacteria which can be pathogenic [118,119]. *Bacillus* species are ubiquitous, found in soil and the healthy flora of many organisms, including animals and plants [120]. Pathogenic *Bacillus* species include *B. anthracis* [119] (anthrax) and *B. cereus* [118,121]. *B. anthracis* is well known as a threat for bioterrorism and has been employed as a bioweapon in the past [122]. *B. cereus* is a common agent of food poisoning, producing cereulide, a potent emetic toxin [123], which is rapid-acting and capable of causing vomiting within hours of consumption of contaminated foods. *B. subtilis* is a common benign soil organism [124], which is motile by peritrichous flagella [125]. Numerous flagellotropic bacteriophages infecting the *Bacillus* species exist [126,127,128,129,130,131], with PBS1 being the most well known. PBS1 was the second flagellotropic phage ever discovered and determined to be flagellum-dependent, following χ [126,127,132].

PBS1 is a giant *Myoviridae* phage of *B. subtilis*, attaching to the flagellum via multiple (typically 3) corkscrew-shaped tail fibers [132,133]. Similarly to χ, PBS1 requires flagella to be functional in addition to simply being present for infection to proceed [127,132]. However, PBS1 is capable of binding to paralyzed flagella, unlike χ [132]. The PBS1 genome is large, with a size of 252,197 bp (NCBI GenBank accession MF360957.1) [134]. Interestingly, the genome is double-stranded DNA, but thymine is completely replaced with uracil [135]. PBS1 has an icosahedral capsid with a diameter of 120 nm, a tail 200 nm in length, and approximately 125-nm-long helical tail fibers [133]. PBS1 also notably displays “contraction fibers,” which are visibly sticking out from the tail sheath only when the tail is contracted [133].

### 3.3. Agrobacterium Phage 7-7-1

*Agrobacterium*, particularly *A. tumefaciens*, also referred to as *A. fabrum*, *A. radiobacter*, or *Rhizobium radiobacter*, is a well-studied Gram-negative plant pathogen, the causative agent of crown gall disease [136]. It has also been shown to be an opportunistic human pathogen in extraordinarily rare cases [137,138]. *A. tumefaciens* additionally finds a practical use in the genetic manipulation of plant cells [139].

*Agrobacterium* phage 7-7-1 was discovered in 1977 and determined to be flagellotropic [140]. This virulent *Myoviridae* phage infects *Agrobacterium* sp. H13-3, formerly known as *Rhizobium lupini* H13-3 [141], a close relative to the well-studied plant pathogen *A. tumefaciens*. Phage 7-7-1 possesses a 69 nm icosahedral head, short 16 nm tail fibers, a 127 nm contractile tail [142], and putative head fibers [143]. Its 69,391 bp genome consists of 127 open reading frames [142]. Phage 7-7-1 has been shown to utilize LPS as a secondary receptor [68]. It is hypothesized that the phage attaches to LPS and depolymerizes it to reach the surface of the cell [68,143].

### 3.4. Caulobacter Phage ΦCbK

*Caulobacter crescentus* is a unique bacterial species as it has two distinct cellular phenotypes: stalked and swarmer cells [144,145,146]. Replication of a stalked *C. crescentus* cell will produce one stalked cell and one swarmer cell. Stalked cells attach to surfaces via stalks adhered to the surface by a powerful adhesive holdfast [144,145,147]. Swarmer cells are motile by a single polar flagellum and eventually differentiate into immotile stalked cells. *C. crescentus* is a generally non-pathogenic Gram-negative oligotrophic aquatic organism known for growth in freshwater [146]. *Caulobacter* bacteria are only pathogenic in rare cases [148].

Phage ΦCbK is a large, corndog-shaped flagellotropic *Siphoviridae* phage infecting *C. crescentus* [149,150]. It has a 275–300-nm-long non-contractile tail, and an oblong head measuring 195 by 64 nm [133,151]. It possesses head fibers and a short tail fiber. ΦCbK is a virulent phage and has a large genome with a size of 205,423 bp and with 319 protein-encoding open reading frames [152]. Phage ΦCbK is only capable of infecting swarmer cells and has thus been used in the past to characterize the process of the differentiation of cells into the two cell types [153]. The flagellotropic phage lifestyle of ΦCbK is especially advantageous for the infection of *C. crescentus*, because only newly divided cells produce flagella. This increases the likelihood that the phage will infect a healthy cell that recently arose from cell division, rather than wasting effort by binding to non-fit cells. The secondary receptor for ΦCbK is known to be a Type IV pilus portal complex on the cell surface [154]. This complex is required for irreversible binding and DNA entry. The current hypothesis is that ΦCbK uses its head fibers and the rotation of the flagellum to reach the cell’s surface, where it then interacts directly with the pilus portal protein [154].

### 3.5. Campylobacter Phage F341

*Campylobacter jejuni* is a pathogenic bacterium that is a common cause of bacterial gastroenteritis, known particularly for contaminating poultry products [155,156,157]. Campylobacteriosis can be fatal in rare cases [158,159]. *C. jejuni* is a microaerophilic, helical, motile bacterium, which swims using a single polar flagellum or two polar flagella [155,156,157]. F341 is a *Myoviridae* phage of *C. jejuni* which has been proven to be flagellotropic [160]. F341 cannot bind to cells that lack flagella or have paralyzed flagella [160]. TEM images clearly show F341 virions attached to the flagellar filament via short tail fibers [160], appearing very distinct from the longer tail fibers mediating the interaction between the host flagella and χ or PBS1 [87,132], or the head fibers seen with ΦCbK and possibly 7-7-1. The genome of phage F341 has not been sequenced.

### 3.6. Pseudomonas Phage ΦCTX

*Pseudomonas aeruginosa* is a Gram-negative, strictly aerobic, rod-shaped pathogenic bacterium, which is well known for its multi-drug resistance [161,162]. It causes nosocomial infections, particularly in immunocompromised patients. *P. aeruginosa* pneumonia is a very common reason of death in persons afflicted with cystic fibrosis (CF) [163,164]. CF is a genetic condition which results in the buildup of mucus in the lungs, leading to breathing difficulties and a reduced life expectancy of 40 to 50 years. *P. aeruginosa* can form strong biofilms in the lungs of CF patients [165], which are very difficult to eradicate. Compounded with *P. aeruginosa*’s natural antibiotic resistance phenotype, these biofilms can be nearly impossible to treat with antibiotics [165]. Phage therapy has been explored for treatment of *P.*-*aeruginosa*-associated infections in human patients as a last resort emergency treatment [50].

ΦCTX is a flagellum-dependent phage infecting *P. aeruginosa* [166]. This small *Myoviridae* phage has a DNA genome 35,583 bp in length with 47 open reading frames (NCBI GenBank accession number Y13918) [134]. This phage has been shown to attach to flagella [166]. Phage infection can be blocked by the addition of flagellin antisera, or antisera specific to the 91–116 and 100–116 peptides. Furthermore, the addition of the peptides themselves blocks infection [166].

### 3.7. Other Flagellotropic Phages and Their Hosts

*Proteus* is a genus of Gram-negative, facultatively anaerobic, enteric bacteria that is opportunistically pathogenic [167,168,169]. *Proteus* bacteria are highly motile by peritrichous flagella and are known for their robust swarming motility phenotype [170,171]. A flagellum-dependent phage named PV22 has been discovered infecting *P. vulgaris* [172]. Interestingly, this phage is capable of binding to *C. jejuni* flagella, although it cannot infect and produce progeny [172].

A transducing phage, PhiOT8, has been found to be flagellotropic. The host range of this phage is surprisingly broad, as it can infect *Serratia* sp. ATCC39006 and *Pantoea agglomerans* [173]. Both are members of the *Enterobacterales* order, but the *Serratia* and *Pantoea* genera are part of two distinct families, *Yersiniaceae* and *Erwiniaceae*, respectively [174,175]. This is one of the few examples of a flagellotropic phage infecting bacteria across multiple families, another example being phage χ, infecting members of the *Enterobacteriaceae* and *Yersiniaceae* families [87,115].

There have been numerous phages that were determined to be χ-like by sequence homology and assigned to the genus Chivirus [176,177,178,179,180,181,182]. Only a select few of these have been explored further. Phage YSD1 is a much more recently discovered χ-like phage, which also infects Salmonella enterica [180]. It is most known for infecting the highly pathogenic S. enterica serovar Typhi, the causative agent of typhoid fever. YSD1 shares 97% nucleotide sequence identity and 99% amino acid sequence identity with χ, and likely follows a similar infection pathway [180]. Many other phages have been putatively determined to be members of the χ-like cluster, but only a select few have been positively determined to be flagellotropic. The Escherichia phage Utah shows 90.4% nucleotide sequence identity to χ and has been determined to be flagellotropic by testing targeted deletions abolishing motility in the host [177]. The phage iEPS5, also infecting Salmonella, has been more definitively proven to be flagellotropic via electron microscopy [179].

Numerous relatives to PBS1 exist, the most well-known being AR9 [129,183,184], which is used as a general transducing phage in *B. subtilis*. Phages SP3 [130] and PBP1 [131] also infect *Bacillus* via flagella.

The *Agrobacterium* phage Milano shares 89.1% amino acid identity to 7-7-1 [142,185], and likely follows a similar infection pathway [185]. The major distinction is that Milano infects the plant pathogen *A. tumefaciens*, rather than the benign *Agrobacterium* H13-3. *A. tumefaciens* phages GS2 and GS6 are also homologous to 7-7-1, and they have been shown to attach to flagella via electron microscopy [186].

*Caulobacter* phages ΦCb13 and Φ6 are closely related to ΦCbK and likely also infect *C. crescentus* via the flagellum [149,187]. More recently, five other *C. crescentus* phages were discovered and characterized as related to ΦCbK: CcrMagneto, CcrSwift, CcrKarma, CcrRogue, and CcrColossus [188].

## 4. Overall Infection Process and Interactions with Flagella

### 4.1. Requirements for Adsorption into the Bacterial Flagellum

Requirements for phage adsorption into bacterial flagella are surprisingly diverse among the handful of well-studied flagellotropic phages. While some phages merely require the presence of a flagellum, *Salmonella* phage χ has strict requirements for adsorption, as it requires flagella to be present, functional, and capable of rotating CCW [87,116]. The *B. subtilis* phage PBS1 has a similar interaction, although its requirements are less strict as it has been shown to be capable of adsorbing into the flagella of *Bacillus* protoplasts [132]. Bacilli protoplasts generally retain their flagella, although torque generation is impossible without the cell wall, which is required for the anchoring of the stator subunits. While PBS1 can adsorb into cells with paralyzed flagella, this is not the case for χ and other flagellotropic phages including the *Agrobacterium* sp. H13-3 phage 7-7-1 [88]. The interaction between the phage and flagellum is very complex and barely understood. While the phage must adsorb into the filament strongly enough to remain attached during the flagellum’s vigorous rotation, overly strong attachment would block translocation to the cell surface.

A very important question to answer is what characteristics of the flagellar filament mediate binding by flagellotropic phages [89,90]. Many flagellotropic phages can infect multiple species with very little conservation in the variable flagellin D2 and D3 domain structure [87,115,173]. Even different flagellin homologs within the same organism show significant variation. Despite this, most flagellotropic phages are able to bind to all flagellin homologs produced by their respective hosts. For instance, phage χ can adsorb effectively to flagellar filaments composed of either FliC or FljB *in Salmonella* Typhimurium [189]. *C. crescentus* has six flagellin proteins: FljJ, FljK, FljL, FljM, FljN, and FljO, and flagellotropic phage ΦCbK is capable of binding to all six flagellins, although the adsorption efficiency of phages into flagellar filaments lacking particular flagellin proteins varies [187]. These flagellins must therefore have a common feature that mediates binding. Phage 7-7-1 only infects the *Agrobacterium* sp. H13-3, but not the closely related *A. tumefaciens*. This may be due to differences in their flagellin structure. Similarly, χ is capable of binding to *Salmonella* Typhimurium flagella and infecting the cells, but cannot bind to or infect those of the related serotype Enteritidis [112]. This flagellar structural distinction remains mostly elusive. Phage χ was shown to not be capable of infecting *S. enterica* serotypes expressing the flagellin “g” antigen [112]. However, the precise motifs determining this antigenic characteristic are not known, and certain flagella not containing “g” antigenic flagellin may not allow χ binding either. This is further complicated by the fact that χ can bind, albeit poorly, to *E. coli* cells producing polyhooks, which are extended hook structures without a filament [116] due to a deletion in the hook molecular ruler gene *fliK* [190].

### 4.2. After Adsorption: Translocation to the Cell Surface

While adsorption requirements vary among flagellotropic phages, the requirement of flagellar rotation for infection is absolute. The now commonly accepted theory of the mechanism of flagellotropic phage translocation along the flagellum is known as the “nut and bolt” model [116], which was originally established by Howard Berg for phage χ, and has been since applied to other systems. This theory asserts that χ wraps its single tail fiber around the flagellar filament, using the rotation to reach the cell surface to continue infection. This is supported by data showing that χ is incapable of infecting not only cells with paralyzed flagella, but also chemotaxis-deficient cells that rotate their flagella only CW [116]. While not all flagellotropic phages possess a single tail fiber [132,140,185], it is believed that this mechanism of infection is largely conserved, although the RBPs themselves are highly diverse in structure. The *Bacillus* phage PBS1 binds to its host’s flagellum via tail fibers [126,132], which have been visualized by transmission electron microscopy (TEM) in the 1960s as wrapped around the *Bacillus* flagellum, similarly to the appearance of χ attached to flagella. The structure of the χ-like phage YSD1, infecting the *Salmonella* ser. Typhi, is comparable to χ [180]. This phage likely follows a similar infection mechanism to χ, although this has not been extensively explored.

**Figure 1:**
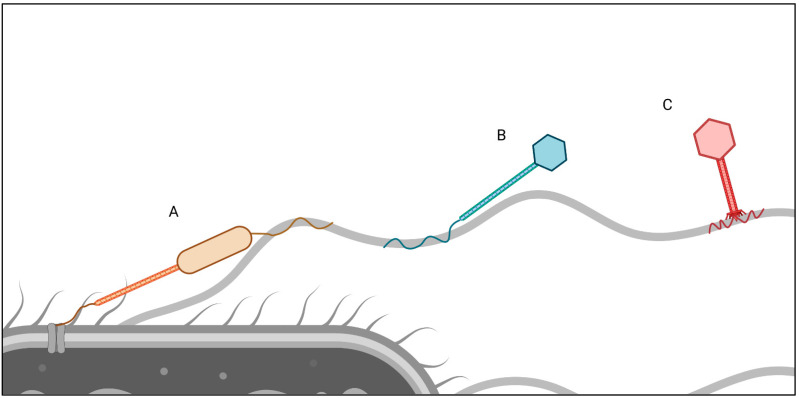
Flagellotropic bacteriophages and their diverse mechanisms of adsorption into bacterial flagella. (A) ΦCbK and its relatives attach to the *Caulobacter crescentus* flagellar filament via their head fibers and interact with their cell surface receptor using their tail and tail fiber [154]. These interactions may occur simultaneously. (B) *Salmonella* phage χ and its relatives attach to their host flagella with their single, long tail fiber [87,116,180]. (C) *Bacillus* phage PBS1 and its relatives use their multiple corkscrew-shaped tail fibers to attach to flagella [132]. Created with BioRender.com (accessed on 17 June 2022).

The mechanisms of phage binding to flagella are very diverse (Figure 1). Certain phages have been described to produce capsid fibers, which may mediate the interaction with the flagellum [143,154]. As an example, ΦCbK possesses head fibers, which mediate the interaction between the phage and the flagellum [154]. Early publications showed via electron microscopy that 7-7-1 virions were found in close proximity to the flagellum, but were not in contact with the flagellum via their short tail fibers [140]. This resulted in the theory that the association with the flagellum is weak, and phages had detached from the filament during grid preparation. As electron microscopy technology has improved over the decades, fine fibers have been observed protruding from the capsid of 7-7-1 [143].

## 5. Applications

### Specific Applications for Flagellum-Dependent Phages

The aforementioned forced evolutionary tradeoff imparted upon a motile pathogen by a flagellotropic phage makes these niche phages attractive for clinical use. Numerous pathogens rely on motility for virulence [51,52,191], including some that are susceptible to known flagellotropic phages such as those of the genera *Campylobacter*, *Escherichia*, and *Salmonella*. Bacteriophage χ, in particular, depends on the host antibiotic efflux complex AcrABZ-TolC during its infection cycle [65]. This means χ would potentially force two distinct evolutionary tradeoffs onto a pathogen: if the cell reduces its production of flagellar components, the virulence would be attenuated, and if the cell reduces expression of components of the AcrAB/TolC efflux pump, it would become more susceptible to the antibiotic substrates of the pump. The *Agrobacterium* phage 7-7-1 requires LPS for its infection process as a secondary receptor [68]. LPS is an important virulence factor in a number of Gram-negative human pathogens [192,193,194,195,196,197,198,199]. LPS, in addition to flagellin, is an incredibly potent antigen recognized by the human immune system. Since secondary receptors of flagellotropic phages in general have not been thoroughly explored, it is reasonable to predict that a clinically relevant flagellotropic phage also requires LPS. Such a phage would impose evolutionary tradeoffs for both the LPS and flagellar systems.

The important plant pathogen *A. tumefaciens* and the related *Agrobacterium* sp. H13-3 are infected by flagellotropic phages Milano and 7-7-1, respectively [140,185]. *A. tumefaciens* is the causative agent of crown gall disease in numerous plants, and various steps are taken by farmers to avoid infecting their crops with *A. tumefaciens* [136,200,201]. This organism uses chemotaxis and motility as virulence factors to colonize plants [202,203]. Once a plant is infected, it is difficult to cure the infection; thus, preventative medicine is the technique of choice [201]. One common method for protecting against crown gall disease is the inoculation of plants with a non-pathogenic *Agrobacterium* strain, which outcompetes pathogenic *A. tumefaciens*, largely through the production of a bacteriocin [201]. A similar technique could be used with a bacteriophage. A flagellotropic phage may be of particular interest, considering the importance of motility for *A. tumefaciens* to infect plants. Phage could be applied to plants indiscriminately, as a phage such as 7-7-1 would have no effect on humans if consumed, in contrast to antibiotic treatment.

Flagellotropic phages have been applied as a tool to identify motility genes [204]. By infecting a transposon mutant library with a flagellotropic phage, most of the resulting surviving mutants will have knockouts of genes involved in motility, which can then be studied. ΦCbK has been used to characterize cell differentiation in *C. crescentus* [153]. Specifically, *C. crescentus* mutants deficient in the ability to differentiate into swarmer cells would be entirely resistant to ΦCbK.

Phage PhiOT8 has been characterized as a general transducing phage for *Serratia marcescens* and *Pantoea agglomerans* [173]. This is highly useful, because transducing phages that work across different families are rare [205]. Likewise, the *Bacillus* phages PBS1 and AR9 have been used for transduction [129].

Purified phage RBPs binding flagella have been shown to inhibit growth in their respective host bacteria even when viable phage particles are not present. This has been demonstrated with the FlaGrab protein of phage NCTC 12673, which inhibits growth in *Campylobacter jejuni* after binding to flagella [206,207,208]. More recently, the putative RBP Gp4 of *Agrobacterium* phage 7-7-1 has been presented to inhibit growth in its host [143].

## 6. Significant Gaps in Knowledge and Directions for Future Research

### 6.1. In Vivo Research

Relative to their potential applications, bacteriophages are generally understudied, and this is even moreso true for flagellotropic phages. Much knowledge about phages has been gathered through a handful of phages, while most of the billions of phages remain unstudied. Due to the forced evolutionary tradeoff imparted by flagellotropic phages, they are of particular interest for phage therapy. Marketing a phage as a curative agent in humans requires well-defined knowledge regarding said curative agent [44,209,210]. This is no different for a phage as it is for an antibiotic compound. A significant gap in flagellotropic phage research is in vivo experimentation. The hypothesis regarding the evolutionary tradeoff is a valuable theory. However, to validate the use of flagellotropic phages for antimicrobial applications, the practicality of this tradeoff must be evaluated. The main issue arises from the overall lack of research surrounding flagellotropic phages. The most well-studied flagellotropic phages are PBS1, χ, 7-7-1, and ΦCbK. Of these, only χ infects a human pathogen [106,108], making it suitable for healthcare applications. Milano, a 7-7-1 relative, could be used in vivo in a plant model to control crown gall disease caused by *A. tumefaciens*. Another consideration is the use of “phage cocktails” consisting of multiple phages, because this vastly reduces the chance that the bacterium develops resistance to the treatment [211,212,213,214]. Including a flagellotropic phage in a phage cocktail could certainly be advantageous; however, before a flagellotropic phage could be included in an antimicrobial cocktail, more simple in vivo experimentation must be conducted on models such as mice [215], *Caenorhabditis elegans* [216], or *Galleria mellonella* [217,218].

### 6.2. The Role of RBPs and Flagellin in Determining Host Range

A phage receptor is the cellular structure in the bacterial host that the bacteriophage binds to to initiate infection. The primary receptor of flagellotropic phages, by definition, is the flagellum. Some more common phage receptors include LPS [219,220], outer membrane channels [221,222], as well as different cellular appendages such as pili [223,224]. The phage RBP is the phage component that binds to the receptor [225]. For instance, the primary receptor of flagellotropic phages is the flagellum, and the RBP of phages χ and PBS1 is the tail fibers [87,132], while for ΦCbK and possibly 7-7-1, it is the head fibers [143,154]. Since the closely related phages χ and YSD1 have different host ranges within the *S. enterica* serotypes, there must be differences within the phage genomes that determine this host range. A possible aspect could be variations within the tail fiber protein, but this is not necessarily the only factor. Subtle differences in the baseplate or tail itself could alter the binding characteristics of these respective phages. In phages such as ΦCbK and 7-7-1, which possess head and tail fibers, these may act as independent RBPs. From the current evidence, it is likely that the head fibers mediate the interaction with the flagellum, while the tail fibers interact with the secondary receptor [154]. This confounds the issue, as it is likely that each RBP plays a role in determining the host range.

Experimentation towards the investigation of phage–host interactions is complicated by the inherent difficulty in applying targeted mutagenesis to virulent phages [226,227]. Thus, a directed evolution approach using random mutagenesis may be the method of choice. Both ultraviolet light and chemical mutagens have been employed for phage mutagenesis [228,229,230,231]. By incubating mutagenized flagellotropic phages with a bacterium closely related to its host organism, it may be possible for host range mutants to arise. These could then be isolated and their genomes sequenced to identify mutations that lead to a change in the host range.

In addition to the RBP itself, the flagellin structure affects the host range [112,166]. This has not been explored in-depth, apart from the identification of *P. aeruginosa* flagellin motifs mediating the attachment of phage ΦCTX [166]. It is not known whether flagellin structural motifs determining a host’s susceptibility to a flagellotropic phage are inclusionary or exclusionary in nature. “Inclusionary” would imply that a particular motif or set of motifs must be present in the flagellar filament for adsorption to occur. “Exclusionary” would imply that particular motifs block adsorption by a flagellotropic phage, and that these structures must simply be absent for binding to occur.

### 6.3. Translocation to the Cell Surface

Flagellotropic phages have been studied generally for their unique flagellum-dependent nature. However, just like any other phage, the infection process is more complicated than the initial attachment to the primary receptor. The aforementioned “nut and bolt” model [116] is the prevalent hypothesis of the mechanism by which flagellotropic phages reach the cell surface, although this translocation has not been visualized. Important facets of this interaction are also unknown, such as the flagellin structural requirements for adsorption, the precise rate of phage translocation along the filament, the molecular mechanism of the tail fiber wrapping around the filament, the transition between attachment to the flagellum, and the interaction with a cell surface component for ejection of DNA into the host cell. Observing phage translocation is a challenge, as phages are generally too small to be seen with typical light microscopy [232]. The use of electron microscopy to see biological processes occurring in living systems in real time is a major challenge. Techniques such as liquid-cell electron microscopy may make viewing phage infection in situ more viable in the future [233,234,235,236]. Newer, more advanced techniques such as super-resolution microscopy could be employed to observe this interaction [237,238].

The interaction between the phage and the bacterial flagellum is transient yet strong. As previously stated, a rotating flagellum is always required for flagellotropic phage infection, and in most cases even for adsorption [116,132]. It is reasonable to assume that the attachment to the secondary receptor at the cell surface represents the stronger interaction. When phages reach the cell surface, they would then be irreversibly bound, while phages on the flagellum are only transiently associated. The wrapping of the tail fiber(s) or head fiber(s) around the filament may also rely on rotation to twist the fiber into the filament grooves to initiate stronger binding.

The transient nature of the flagellum–phage interaction presents technical difficulties in studying this process. Since flagellotropic phages generally require a rotating flagellum for attachment to occur, in vitro methods to study protein–protein interactions such as co-immunoprecipitation, isothermal titration calorimetry, and surface plasmon resonance will likely be ineffective. Chemical crosslinking combined with mass spectrometry may prove to be an effective tool, as it allows for the capturing of transient interactions between proteins through the formation of covalent bonds between associated proteins [239,240], which has been used to study phage f1 [241]. Alternatively, phage and live motile cells could be mixed, followed by the addition of a chemical crosslinker. This could covalently bind the phage to the flagellar filament, which could then be sheared and purified for subsequent analysis. Electron microscopy and mass spectrometry could then be applied to identify the interaction partners [242,243,244,245].

### 6.4. Secondary Receptors and other Required Cellular Components

Due to the complexity of phage infection processes, the flagellum is not always the only target of flagellotropic phages. Phage 7-7-1, for instance, uses LPS as a secondary receptor [68,143]. It is thought that 7-7-1 depolymerizes LPS to reach the cell surface to facilitate DNA injection. Phage χ requires the presence of the AcrAB/TolC multi-substrate efflux system in its host *Salmonella* [65]. This complex may serve as a secondary receptor, although the precise purpose of AcrAB/TolC during the χ infection cycle is unknown. The *Caulobacter* phage ΦCbK utilizes a pilus portal protein as a secondary receptor on the cell surface [246]. The requirement for these secondary components begs a very important and puzzling question: why are flagellotropic phages generally unable to bind directly to their secondary receptors? Even if the mechanism of DNA entry simply lies at the base of the flagellum, the phage should be able to infect its host, although with lesser efficiency, if the flagellar filament is absent but the basal body is intact. A possible explanation for this phenomenon is that the phage may have to interact with the flagellum and its secondary receptor simultaneously. As a consequence, the phage would possess two RBPs, one for each receptor. Alternatively, the affinity of the phage to its secondary receptor may be weak compared to other, non-flagellotropic phages. The flagellum may be required for the virus to position itself for binding to receptors on the cell surface. Lastly, it is possible that some secondary receptors for flagellotropic phages are localized near the flagellar basal body in the cell envelope, thus increasing the likelihood for the phage to bind to its surface receptor. This seems to be the case for the interaction of ΦCbK with the pilus portal protein [246]. Due to the presence of head and tail fibers, phages such as 7-7-1 and ΦCbK may be able to bind to the flagellum and the secondary receptor at the same time. The DNA entry process for χ and PBS1 may be more complicated, because these phages have been proven to bind to the flagellum using their tail fibers [87,116,126,132]. Phage χ, for instance, would have to transition from being attached to the flagellum to attaching to TolC if AcrAB/TolC is the cell surface receptor [65].

The flagellotropic nature of these phages is their hallmark characteristic, but this has distracted researchers from exploring the nuances of these phages’ interactions with motility-independent features of their respective host bacteria. This is magnified by the fact that many flagellotropic phages were discovered around a half-century ago [112,132,140,151], when knowledge regarding flagella and motility was limited. At the time, the flagellum-dependent nature of these phages was the main important distinction from other phages that warranted investigation and also posed a useful way to identify motility genes [204] or differentiation processes in *C. crescentus* [151]. Now that flagellar motility and chemotaxis are well understood, it is time for detailed investigations of flagellotropic phages. Genes required for phage infection can be identified trivially by high-throughput barcoded transposon library screening [247]. However, a significant problem is the dominant identification of motility genes and other genes with pleiotropic impacts on motility [248,249,250]. Alternatively, a library can be specifically selected for motile cells. This has been employed successfully in χ [65], but was only possible through the individual screening of mutants, rather than a high-throughput technique such as Tn-seq.

### 6.5. Discovery and Categorization of Novel Flagellotropic Phages

The general isolation of phages infecting specific hosts from an environmental source is straightforward [251,252,253]. A phage is identified, and subsequent steps can be taken to determine its receptor. Isolating phages by receptor rather than by host is more difficult. While bacterial lineages can be determined relatively trivially by analysis of 16S rRNA [254,255,256], this is more difficult in phages due to the lack of a similar unifying gene. Thus, the classification of phages into different groups, families, or species is often determined through sequence homology and phenotype [257]. There is no well-defined cutoff for the percent of amino acid identity, which categorizes a phage as “χ-like.” For this reason, some *Chivirus* phages share approximately 99% amino acid identity with χ itself [180], while others are much more distant and should not necessarily be categorized as “χ-like” [176,178,181].

## 7. Concluding Remarks

Flagellotropic phages fulfill a very specific niche. The flagellum is a long appendage, making it comparably easy to attach to compared to cell surface components. The presence and proper function of flagella are a general indicator of cell fitness due to their high energy cost and tight regulation [100,102], ensuring a phage is infecting a capable host [103,104]. Flagella are advantageous for bacteria in many ways [82], and therefore repression of motility to avoid infection by a flagellotropic phage has a negative fitness effect on the host. While a certain degree of base knowledge exists about flagellotropic phages, there are currently many more questions than answers. Information about the full lifecycle of flagellotropic phages is a series of incomplete stories. As we approach a post-antibiotic era, phage therapy has great potential to become a helpful tool in combating pathogenic bacteria [15]. Despite the challenges, the unique nature of flagellum-dependent phages makes them excellent candidates for antimicrobial applications (Table 1).

**Table 1:** Flagellotropic phages, verified hosts, and putative secondary receptors. The primary receptor of all the phages listed is the flagellum. Phages are ordered based on their similarity and relative depth of study.

Phage	Host Bacteria	Putative Secondary Receptor(s)	References
χ	*Salmonella enterica* *Escherichia coli* *Serratia marcescens*	AcrAB/TolC	[65,87,112,114,115,116,117,181,258]
YSD1	*Salmonella enterica*	unknown	[180]
Utah	*Escherichia coli*	unknown	[177]
iEPS5	*Salmonella enterica*	unknown	[179]
PBS1	*Bacillus subtilis*	unknown	[87,126,127,128,129,131,132]
AR9	*Bacillus subtilis*	unknown	[129,183,184]
SP3	*Bacillus subtilis*	unknown	[130]
PBP1	*Bacillus subtilis*	unknown	[131]
7-7-1	*Agrobacterium sp.* H13-3	LPS	[68,88,140,142,143]
Milano	*Agrobacterium tumefaciens*	unknown	[185]
GS2	*Agrobacterium tumefaciens*	unknown	[186]
GS6	*Agrobacterium tumefaciens*	unknown	[186]
ΦCbK	*Caulobacter crescentus*	Type IV pili secretion apparatus	[151,152,154,187,188,246]
ΦCb13	*Caulobacter crescentus*	unknown	[149,187]
ΦC6	*Caulobacter crescentus*	unknown	[149,187]
F341	*Campylobacter jejuni*	unknown	[160]
ΦCTX	*Pseudomonas aeruginosa*	unknown	[166]

## Data Availability

Not applicable.
